# Preparative SDS PAGE as an Alternative to His-Tag Purification of Recombinant Amelogenin

**DOI:** 10.3389/fphys.2017.00424

**Published:** 2017-06-16

**Authors:** Claire M. Gabe, Steven J. Brookes, Jennifer Kirkham

**Affiliations:** Division of Oral Biology, School of Dentistry, University of LeedsLeeds, United Kingdom

**Keywords:** recombinant amelogenin, His-tag, nickel column chromatography, protein purification, preparative SDS PAGE

## Abstract

Recombinant protein technology provides an invaluable source of proteins for use in structure-function studies, as immunogens, and in the development of therapeutics. Recombinant proteins are typically engineered with “tags” that allow the protein to be purified from crude host cell extracts using affinity based chromatography techniques. Amelogenin is the principal component of the developing enamel matrix and a frequent focus for biomineralization researchers. Several groups have reported the successful production of recombinant amelogenins but the production of recombinant amelogenin free of any tags, and at single band purity on silver stained SDS PAGE is technically challenging. This is important, as rigorous structure-function research frequently demands a high degree of protein purity and fidelity of protein sequence. Our aim was to generate His-tagged recombinant amelogenin at single band purity on silver stained SDS PAGE for use in functionality studies after His-tag cleavage. An acetic acid extraction technique (previously reported to produce recombinant amelogenin at 95% purity directly from *E. coli*) followed by repeated rounds of nickel column affinity chromatography, failed to generate recombinant amelogenin at single band purity. This was because following an initial round of nickel column affinity chromatography, subsequent cleavage of the His-tag was not 100% efficient. A second round of nickel column affinity chromatography, used in attempts to separate the cleaved His-tag free recombinant from uncleaved His-tagged contaminants, was still unsatisfactory as cleaved recombinant amelogenin exhibited significant affinity for the nickel column. To solve this problem, we used preparative SDS PAGE to successfully purify cleaved recombinant amelogenins to single band purity on silver stained SDS PAGE. The resolving power of preparative SDS PAGE was such that His-tag based purification of recombinant amelogenin becomes redundant. We suggest that acetic acid extraction of recombinant amelogenin and subsequent purification using preparative SDS PAGE provides a simple route to highly purified His-tag free amelogenin for use in structure-function experiments and beyond.

## Introduction

Amelogenesis involves the incremental secretion of an extracellular protein matrix by ameloblasts. Amelogenin, present as a range of related molecules generated through extracellular proteolysis of several alternatively spliced gene products, comprises ~90% of the total extracellular protein matrix and is essential for normal enamel biomineralisation. Amelogenin mutations can lead to amelogenesis imperfecta (AI) in humans (Lagerstrom et al., 1991; Aldred et al., 1992; Lench and Winter, 1995; Collier et al., 1997; Kindelan et al., 2000; Hart et al., 2002; Kim et al., 2004; Wright et al., 2009) and mouse models (Barron et al., 2010) and amelogenin null mice fail to produce enamel (Gibson et al., 2001; Wright et al., 2009; Smith et al., 2016). However, the exact function of amelogenin remains unclear and studies are ongoing to elucidate amelogenin function and the etiologies that underpin AI when the amelogenin gene is mutated.

Our own interest is with the effect of a mouse amelogenin mutation (p.Y64H) on amelogenin aggregation/assembly and ameloblast secretory trafficking. Wild-type (WT) amelogenin begins to self-assemble during transit through the ameloblast secretory pathway (Brookes et al., 2006). On secretion, it forms “nanospheres” of ~25–50 nm in diameter in the developing enamel (Fincham et al., 1995). Ameloblasts are secretory cells that are adapted to handle the large amount of amelogenin transiting through the rough endoplasmic reticulum (ER) and Golgi apparatus (Warshawsky, 1968). This is achieved by utilizing elements of the unfolded protein response (UPR) (Tsuchiya et al., 2008) to maintain ameloblast ER proteostasis; as commonly seen in other secretory cells (Moore and Hollien, 2012). The UPR aids cells to cope with a large secretory load by increasing ER volume and protein folding capacity and increasing the cell's ability to identify and handle misfolded proteins via ER associated protein degradation (Travers et al., 2000). This is important, as up to 30% of proteins destined for secretion can mis-fold spontaneously (Schubert et al., 2000) leading to pathological intracellular protein retention and severe ER stress (Ozcan and Tabas, 2012). Under circumstances when ER stress is abnormally high and prolonged, the UPR switches from a pro-survival mode to a pro-apoptotic mode which leads to cell death (Rutkowski and Kaufman, 2007). Our previous work has shown that the *Amelx*^*p*.(*Y64H*)^ mutation promotes intracellular amelogenin retention, stalls the secretory pathway, and triggers the UPR to switch into pro-apoptotic mode resulting in ameloblast death, thus classifying this case of AI as a conformational disease (Brookes et al., 2014). Our current work seeks to investigate the effect of the p.Y64H mutation on amelogenin-amelogenin interactions. The availability of highly purified WT and p.Y64H amelogenin is therefore essential for use in structure-function studies.

It is possible to purify amelogenin from developing enamel itself but this requires a ready and plentiful supply of secretory stage enamel and expertise in protein purification to isolate a single amelogenin species from the plethora of discrete but related amelogenin molecules present in the tissue that are derived by extracellular processing of several alternatively spliced isoforms (Brookes et al., 1995). This is challenging but the advantage of purifying native amelogenin from tissue over producing recombinant protein in *E. coli* is that the protein will be phosphorylated at serine 16 via translational modification *in vivo* (Takagi et al., 1984; Fincham and Moradian-Oldak, 1993; Fincham et al., 1994) and this is believed to be important to its function (Kwak et al., 2009; Wiedemann-Bidlack et al., 2011). Typically, native enamel proteins are purified from porcine developing enamel as porcine developing teeth can be obtained as a by-product of the meat industry (Aoba et al., 1987; Limeback, 1987). Compared to rodent incisors, each porcine tooth provides a relatively large amount of starting material as the enamel thickness can be up to ten times that of rodent developing tissue. However, pigs must be obtained at a specific age to ensure that developing teeth are in the secretory phase and not all teeth will be undergoing amelogenesis at the same time. The main models for studying amelogenesis, the roles of specific proteins components and any effects of specific mutations are the mouse and rat. Rodents have the advantage that their incisors are continually erupting and the required secretory stage enamel is present in weaned animals of any age. However, the yield of secretory stage enamel per animal is so small that very few published studies have used purified rodent amelogenins.

The need to understand AI pathobiology associated with specific mutations effectively precludes the use of porcine teeth. In addition, as we reported previously in the case of our p.Y64H amelogenin mouse model, there are cases where the mutation results in failure to secrete the affected protein and this in turn prevents our purifying the protein from the enamel itself. The challenge of purifying amelogenin from whole cell extracts would be significant and the only viable source of mutated amelogenin (or for that matter WT rodent amelogenin) is via recombinant technology.

Recombinant proteins are widely used as therapeutic agents and as tools to study structure-function relationships, protein interactions with other molecules and as antigens for antibody production. *E. coli* based expression systems are the most widely used methodology for producing recombinant amelogenin even though post translational phosphorylation of serine 16 will be absent. Baculo virus (Taylor et al., 2006; Xu et al., 2006) and *Leishmania* (Yadegari et al., 2015) expression systems, having the potential to carry out post translational phosphorylation have been used to produce recombinant amelogenin, but as yet do not appear to have been widely adopted perhaps due to uncertainty as to whether the amelogenin was phosphorylated. In contrast, a yeast based expression system has been reported to generate correctly phosphorylated recombinant amelogenin (Cheng et al., 2012) but again has not been widely used. Regardless of the expression system used, the recombinant amelogenin will need to be purified and freed from host cell proteins, amelogenin degradation products, and other contaminants arising from the growth medium. Early reports describing the preparation of recombinant amelogenin used ammonium sulfate precipitation followed by repeated rounds of reverse phase chromatography (Simmer et al., 1994) or ammonium sulfate precipitation followed by cation exchange chromatography and reverse phase chromatography (Ryu et al., 1999) to effectively purify the final product. However, it is now generally more common to purify recombinant proteins by engineering the inclusion of either an N or C-terminal tag comprising of a poly histidine sequence (“His-tag”) or fused to glutathione S-transferase (GST) (Hochuli et al., 1988; Smith and Johnson, 1988). His-tags and GST have a high affinity for immobilized nickel ions and glutathione respectively which allows the recombinant proteins to be purified using affinity column chromatography. Tags typically include a proteolytic cleavage site which allows them to be removed following elution from the column.

In our hands, both His-tagged WT and p.Y64H amelogenins are expressed at acceptable levels in *E. coli* but after nickel column affinity purification they also invariably contain background contaminants especially on silver stained SDS PAGE; an issue also noted by others (Xu et al., 2006). In agreement with Taylor et al. (2006) we also find that the His-tag cleavage is not 100% efficient and our final cleaved product is a mixture of tagged and untagged recombinant proteins. Removal of the His-tag is desirable for functional studies; especially those focusing on intermolecular interactions occurring during amelogenesis in the enamel matrix. That said, the presence of a His-tag did not greatly alter the biological activity of recombinant amelogenin on gene expression in osteoblasts and periodontal ligament fibroblasts compared to extracts of native amelogenin from developing enamel (Svensson et al., 2006; Cheng et al., 2012). Certainly, for our intended applications, the removal of the His-tag is essential to generate unambiguous data in protein-protein interaction studies. Typically, contaminating uncleaved His-tagged protein is removed by a second round of nickel column chromatography. However, due to the histidine content of amelogenin (including a tri-histidine motif; Snead et al., 1985), even untagged amelogenin has a relatively high affinity for nickel, confounding nickel column separation of His-tagged from non-tagged amelogenins.

The aim of the present work was to develop an adjunctive purification step to remove background contaminants from recombinant amelogenins, including uncleaved His-tagged amelogenin, generating amelogenins with a high degree of purity. The results of this work lead us to conclude that we can purify recombinant amelogenin to a high level of purity from *E. coli* without the need for a His-tag using a previously reported acetic acid extraction technique and a single round of preparative SDS PAGE. This provides a faster means of obtaining purified recombinant amelogenin free of any residual His-tag remnants for use in structure-function studies and beyond.

## Materials and methods

### Expression and extraction of recombinant amelogenin

Wild-type amelogenin was expressed using a pET28 expression vector (Novagen, Merck Chemicals Ltd.) modified with a HRV 3C protease site in Rosetta DE3 *E. coli* cells (Novagen). Once the cells reached an optical density of 0.6–1.2 recombinant protein expression was induced by adding isopropyl β-D-1-thiogalactopyranoside (IPTG). Induction of expression was confirmed by analytical SDS PAGE. After shaking overnight incubation at 37°C, the cells were harvested by centrifugation and amelogenin-enriched fractions obtained by extraction in acetic acid as previously reported (Svensson Bonde and Bulow, 2012). In brief, 1 g of pelleted cells were washed by resuspending in 30 mL of 150 mM NaCl. After centrifugation the pellet was resuspended in 30 mL 3% acetic acid, sonicated and heated at 75°C for 20 min. Insoluble material was pelleted by centrifugation and the acetic acid extracts were lyophilized. Lyophilized extracts were dissolved in a minimum volume of 125 mM formic acid and desalted against 125 mM formic acid using size exclusion chromatography (HiPrep 26/10 column, GE Healthcare). The volatile formic acid was then removed by lyophilization.

### Affinity nickel column purification of his-tagged recombinant amelogenins extracted using acetic acid

Lyophilized crude extracts of recombinant amelogenin were dissolved in a minimum volume of nickel column binding buffer (20 mM imidazole, 4 M urea, 50 mM Tris, 400 mM NaCl, pH = 7.6), loaded on to a nickel affinity chromatography column (5 mL HisTrap FF, GE Health Care) up to a maximum protein load of 40 mg and eluted at a flow rate of 4 mL/min. Unbound proteins (column flow through) were collected. Bound recombinant amelogenin was eluted by increasing the imidazole concentration to 200 mM. The recombinant amelogenin was then buffer exchanged into 125 mM formic acid using size exclusion chromatography (HiPrep 26/10 column, GE Healthcare) and the fractions containing recombinant amelogenin collected and lyophilized. Fractions were analyzed by analytical SDS PAGE and anti-amelogenin Western blotting using rabbit IgGs raised against a peptide corresponding to the amelogenin hydrophilic C-terminal (Brookes et al., 2011) as described in the Section entitled Analytical SDS PAGE and Western Blotting.

### His-tag cleavage

The lyophilized recombinant amelogenin, prepared as described above, was dissolved at 2 mg/mL in either 50 mM Tris-HCl, pH = 8 or 0.1 M Na_2_CO_3_, pH = 9 (the Na_2_CO_3_ buffer system was trialed in an attempt to improve cleavage efficiency but also allowed the cleavage products to be directly labeled with fluorescein at this point if required). The His-tag was removed enzymatically by adding recombinant restriction grade protease (HRV3C, Merck) at a concentration of 3 μL enzyme solution per mg recombinant protein. Cleavage was carried out over 20 h at 4°C. The resulting cleavage reaction mixture comprising cleaved recombinant, any uncleaved recombinant, free His-tag and other contaminants was finally buffer-exchanged into nickel column binding buffer by size exclusion chromatography ready for a second round of nickel affinity column chromatography.

### Second round nickel column purification to remove uncleaved recombinants, free his-tag, and cleavage enzyme

A second round of His-tag cleavage was employed to remove cleaved His-tag and other contaminants present in the cleavage reaction mixture including any uncleaved recombinant. Cleavage products in binding buffer were loaded on to the nickel affinity column as described in the Section entitled Affinity Nickel Column Purification of His-Tagged Recombinant Amelogenins Extracted Using Acetic Acid. Unbound proteins (expected to contain the cleaved recombinant amelogenin) were collected in the column flow through. Bound proteins (expected to contain any uncleaved recombinant amelogenin, His-tag, and His-tagged HRV3C) were eluted using a stepped imidazole gradient (50, 60, 70, 90, 205, and 400 mM) in order to optimize the separation. Fractions were analyzed by analytical SDS PAGE as described in the Section entitled Analytical SDS PAGE and Western Blotting.

### Analytical SDS PAGE and western blotting

Analytical SDS PAGE was carried out based upon the method of Laemmli (1970) except 20% v/v glycerol was included in the resolving gel. Proteins were separated using a 12% resolving gel at pH 8.8 and a 4% stacking gel at pH 6.8 using Mini-PROTEAN III or Criterion Cell electrophoresis systems (Bio-Rad laboratories Ltd., Hertfordshire, UK). Samples were dissolved in non-reducing SDS PAGE loading buffer (26 mM Tris-HCl pH 6.8, 10% v/v glycerol, 0.35% w/v SDS, and 0.01% w/v bromophenol blue). Prior to SDS PAGE samples were heated at 90°C for 1.5 min and run at a constant 200 V until the bromophenol blue reached the bottom of the gel. Loading for each gel was optimized in each case by running trial gels.

Following electrophoresis, proteins were detected using Coomassie Blue (Expedeon Ltd., Cambridgeshire, UK) or silver staining (ThermoFischer Scientific, Leicestershire, UK) or were transferred to nitrocellulose membranes (Bio-Rad, Ltd., Hertfordshire, UK) for Western blot analysis. After blocking and washing, the membrane was incubated with primary antibody (rabbit anti-amelogenin teleopeptide; Brookes et al., 2006, diluted 1:2,000 in blocking buffer) for 95 min, washed and then incubated for 90 min with secondary antibody (goat anti-rabbit conjugated to horse-radish peroxidase diluted 1:3,000 in blocking buffer). Immunoreactive protein was visualized using DAB with metal enhancer, (Sigma-Aldrich, Dorsetshire, UK) according to the manufacturer's instructions.

### Alternative purification methodology using preparative SDS PAGE

The cleaved recombinant amelogenin fractions produced by the second round nickel column purification described above still contained traces of uncleaved protein and other minor contaminants when examined using analytical SDS PAGE. To eliminate this problem both rounds of affinity column purification were replaced by preparative SDS PAGE. Desalted and lyophilized cleaved recombinant amelogenin was dissolved in non-reducing SDS PAGE loading buffer (as described in the Section entitled Analytical SDS PAGE and Western Blotting) at a concentration of 5–7mg/1.5 mL. Preparative electrophoresis was carried out using a Model 491 Prep Cell (Bio-Rad Ltd., Hertfordshire, UK). A 12% resolving gel was cast in the 28 mm internal diameter gel tube to a height of 9.5 cm with a 2 cm 4% stacking gel. The gel was run at a constant 12 W power at room temperature using the circulating cooling pump as recommended by the manufacturer. Once the tracker dye reached the bottom of the gel, 2.5 mL fractions were collected at a flow rate of 0.8 mL/min. Fractions of interest were identified by subjecting every third fraction to analytical SDS PAGE. All fractions of interest were then subjected to analytical SDS PAGE to accurately determine any fractions containing cleaved recombinant amelogenin at single band purity on silver staining.

**Figure 1:**
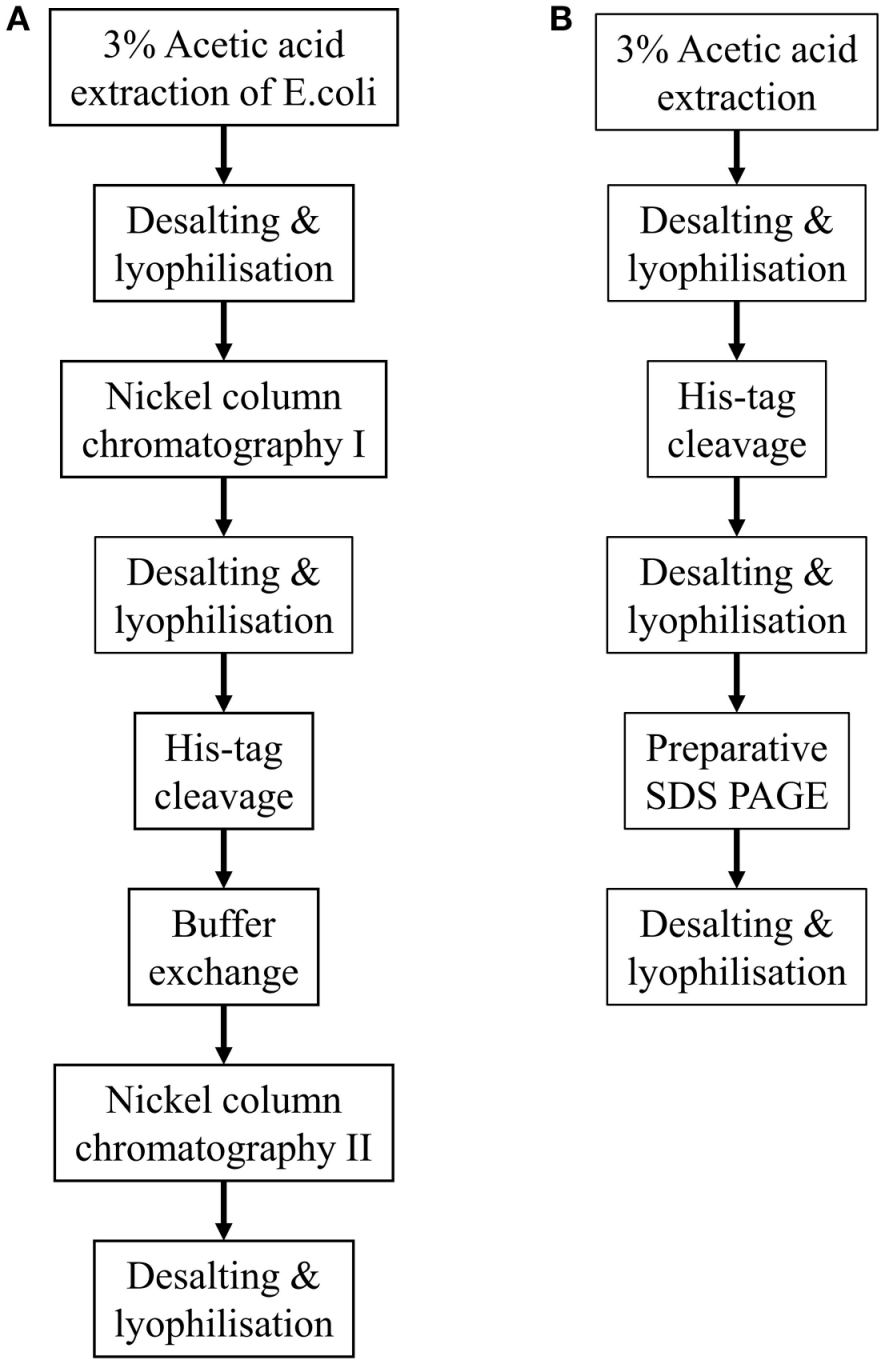
Flow diagrams summarizing the two methodologies described for purifying His-tagged recombinant amelogenin. **(A)** Purification of recombinant His-tagged amelogenin using conventional His-tag column purification. **(B)** Alternative methodology described here using preparative SDS PAGE in place of His-tag column chromatography to purify recombinant His-tagged amelogenin.

The methodologies described above are summarized in Figure 1.

## Results

### Expression and extraction of recombinant amelogenin

**Figure 2:**
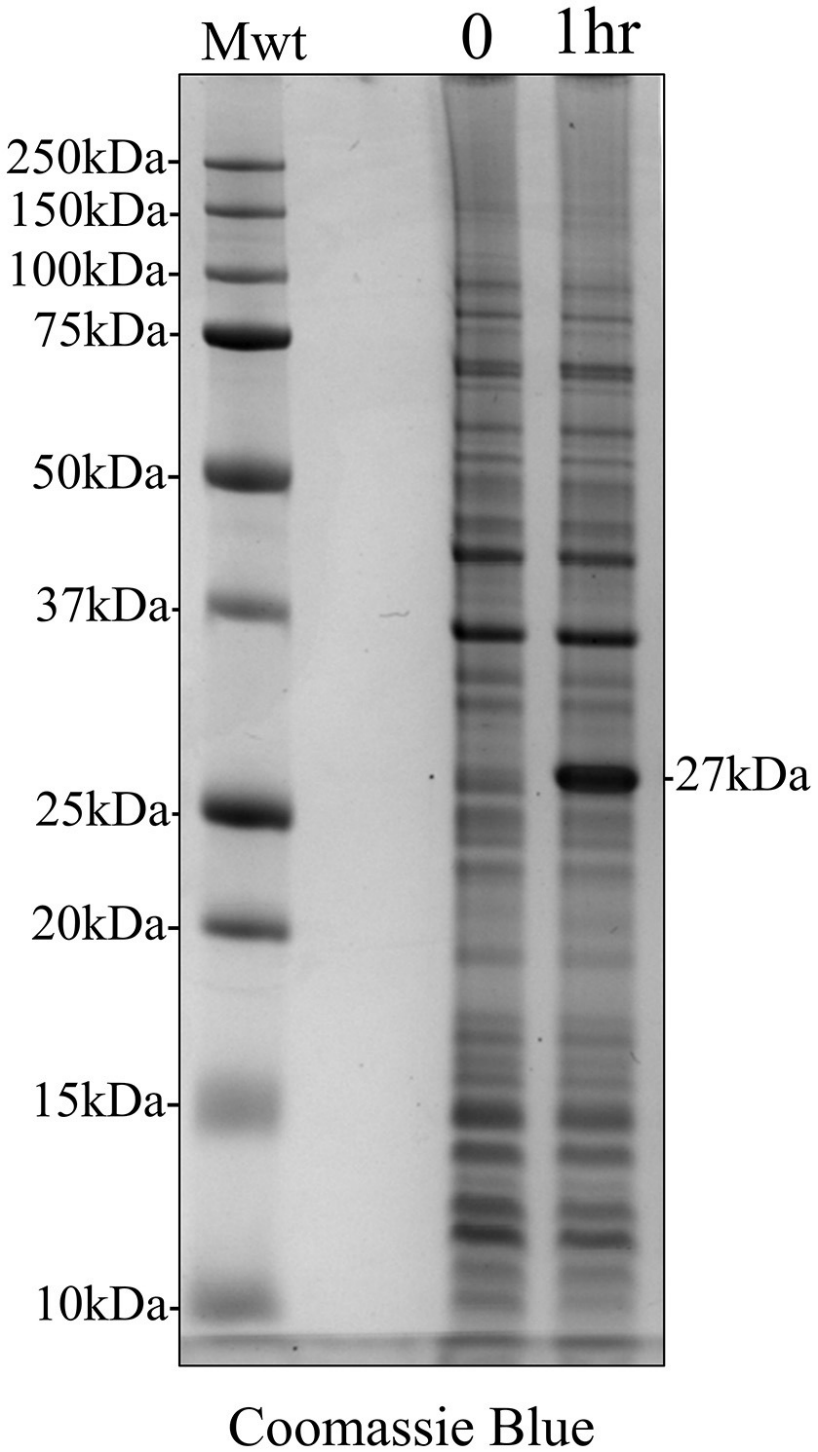
Coomassie Blue stained analytical SDS PAGE showing IPTG induction of recombinant amelogenin expression in *E. coli*. *E. coli* were extracted at the time of induction (0) and 1 h post-induction (1 h). IPTG induced the expression of a 27 kDa protein corresponding to the expected molecular weight of recombinant amelogenin.

Total protein was extracted from *E. coli* both immediately after the addition of IPTG and 1 h post induction to determine the presence of any induced recombinant protein expression using analytical SDS PAGE and Coomassie Blue staining (Figure 2). One hour after IPTG induction, the results clearly demonstrated an additional protein band at 27 kDa, corresponding to the molecular weight of recombinant amelogenin against a background of multiple components derived from the expression system.

### Acetic acid extraction of his-tagged recombinant amelogenin and affinity nickel column purification

**Figure 3:**
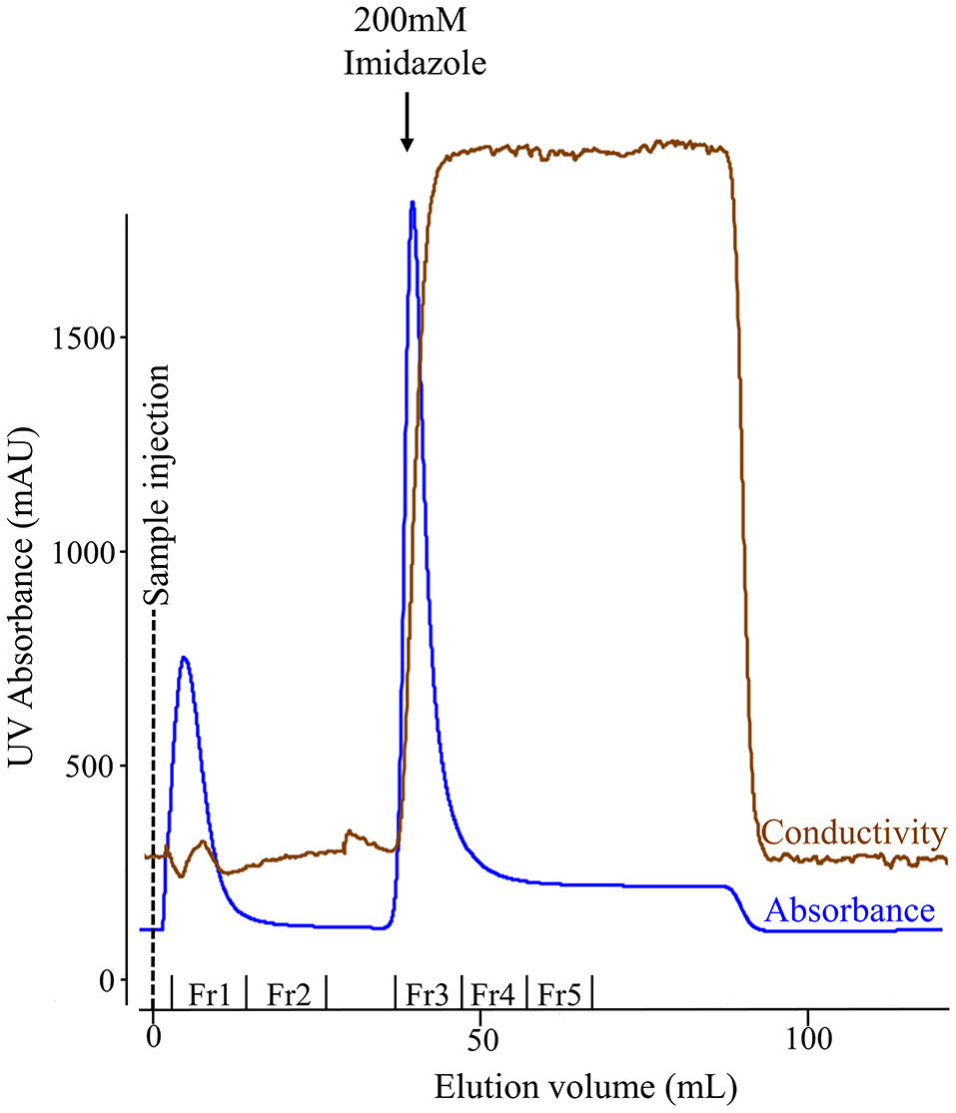
Nickel column chromatography of the soluble protein extracted from *E. coli* using 3% acetic acid. The chromatogram shows fractions comprising proteins that failed to bind the column in 20 mM imidazole (flow-through: fractions 1–2) and bound proteins eluted when the imidazole concentration was increased from 20 to 200 mM (fractions 3–5). The conductivity trace indicates the increase in imidazole concentration in the buffer flowing through the column.

**Figure 4:**
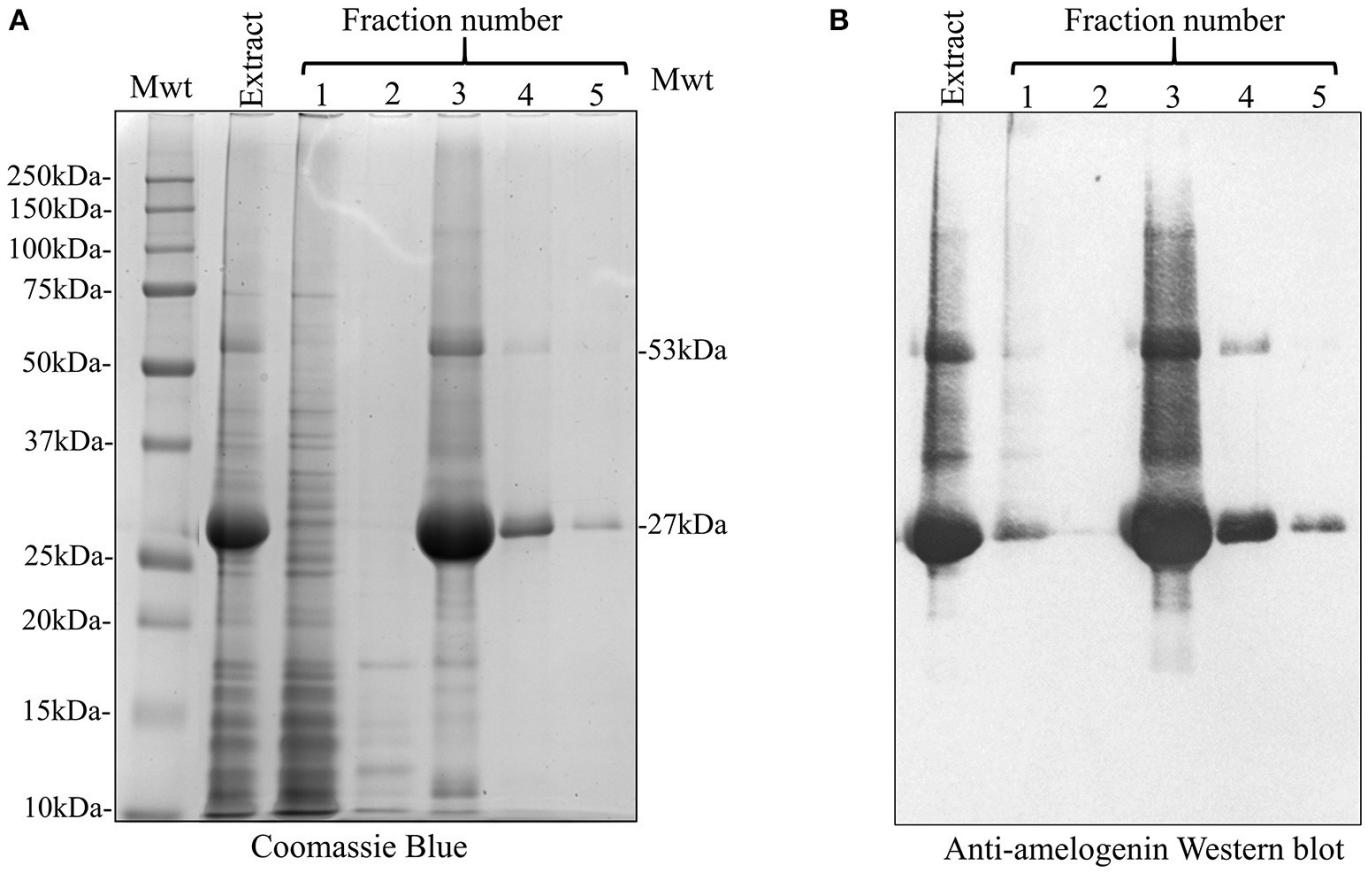
SDS PAGE and anti-amelogenin Western blot analysis of the crude 3% acetic acid *E. coli* extract and nickel column fractions 1–5. **(A)** Extraction with 3% acetic acid (Extract) generated an enriched recombinant amelogenin fraction. In particular, bacterial contaminants migrating above 27 kDa were selectively removed (compare with Figure 2). Fractions 1–2, corresponding to the column flow-through, contained many of the bacterial proteins contaminating the crude acetic acid extract. Fractions 3–4 contain proteins eluted by 200 mM imidazole. The 27 kDa recombinant protein is further enriched but still contains contaminating proteins. **(B)** Corresponding anti-amelogenin Western blot confirms the identity of the 27 kDa recombinant protein as amelogenin. The smear centered on the 53 kDa band correspond to re-equilibrating amelogenin aggregates stable in SDS (dimers at 53 kDa being the predominant species present).

We used affinity chromatography to purify His-tagged recombinant amelogenin following acetic acid extraction of the crude bacterial extracts (Figure 3). Fractions 1–2 (Figure 3, Fr1, Fr2) contained the column flow-through i.e., material that did not bind to the nickel column in 20 mM imidazole loading buffer. Fractions 3–5 (Figure 3, Fr3–Fr5) contained the column bound material eluted by 200 mM imidazole, with the bulk of the material eluting in fraction 3. The fractions were then characterized by analytical SDS PAGE (Figure 4A) and Western blotting using anti-amelogenin antibodies (Figure 4B). Analytical SDS PAGE showed that the original crude bacterial acetic acid extract was clearly enriched with the putative recombinant amelogenin migrating with a relative molecular weight of ~27 kDa but also contained a number of contaminating proteins migrating between 10 and 75 kDa. The majority of these contaminants were not bound by the nickel column and eluted in the flow through (Figure 4A, fractions 1–2). Fractions 3–5, eluted from the column in 200 mM imidazole, contained the putative recombinant amelogenin. The level of contaminants in the putative recombinant amelogenin fraction was reduced after nickel affinity chromatography but some contamination was still evident at this loading. The corresponding anti-amelogenin Western blot confirmed the identity of the 27 kDa protein to be recombinant amelogenin. Anti-amelogenin-immunoreactive protein was also evident at above 27 kDa, as a smear centered on ~53 kDa. This corresponds to the formation and re-equilibration of amelogenin complexes (predominantly dimers) that can exist during SDS PAGE of amelogenin (Limeback and Simic, 1990).

### His-tag cleavage and repeated nickel column purification to remove uncleaved recombinants, freed his-tag, and cleavage enzyme

**Figure 5:**
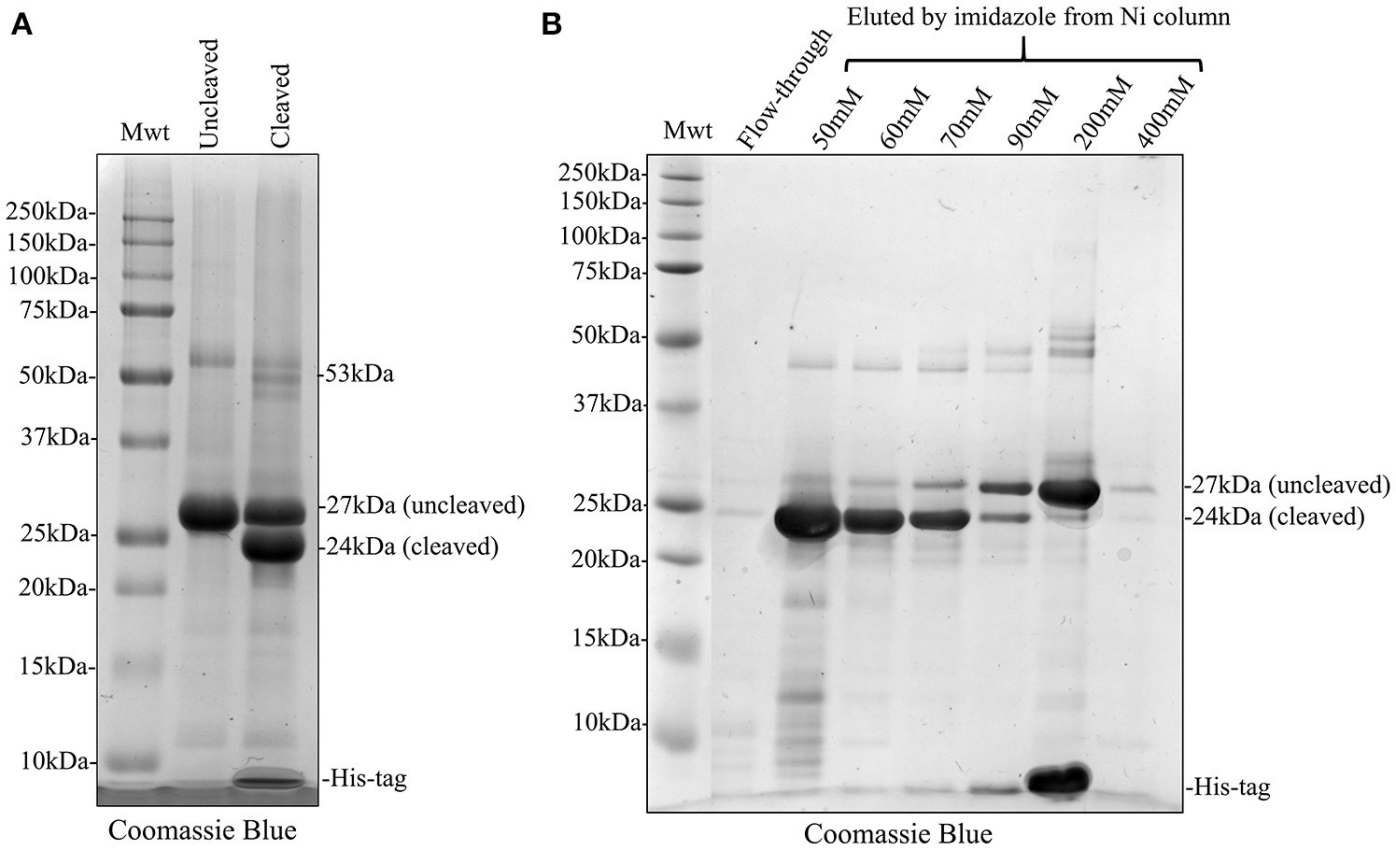
Coomassie Blue stained SDS PAGE showing His-tag cleavage of recombinant amelogenin and second round of nickel column purification using stepped elution gradient to optimize purification of cleaved amelogenin from the cleavage reaction mixture. **(A)** Cleavage of His-tagged 27 kDa recombinant (Uncleaved) with HRV3C protease was not 100% efficient. The cleaved reaction mixture (Cleaved) contained 24 kDa cleaved and 27 kDa uncleaved recombinant. **(B)** The cleaved recombinant was retained on the nickel column despite lacking a His-tag and did not appear in the column flow-through (Flow-through). Subsequent elution with an increasing stepped imidazole gradient eluted the cleaved recombinant at imidazole concentrations of 50 mM and above but some uncleaved recombinant amelogenin and lower molecular weight contaminants were co-eluted. The bulk of uncleaved recombinant amelogenin and free His-tags were eluted by 200 mM imidazole. Elution using 60 mM imidazole was selected since compared to 50 mM this would increase the yield of cleaved recombinant without overly increasing the elution of uncleaved recombinant as a contaminant.

**Figure 6:**
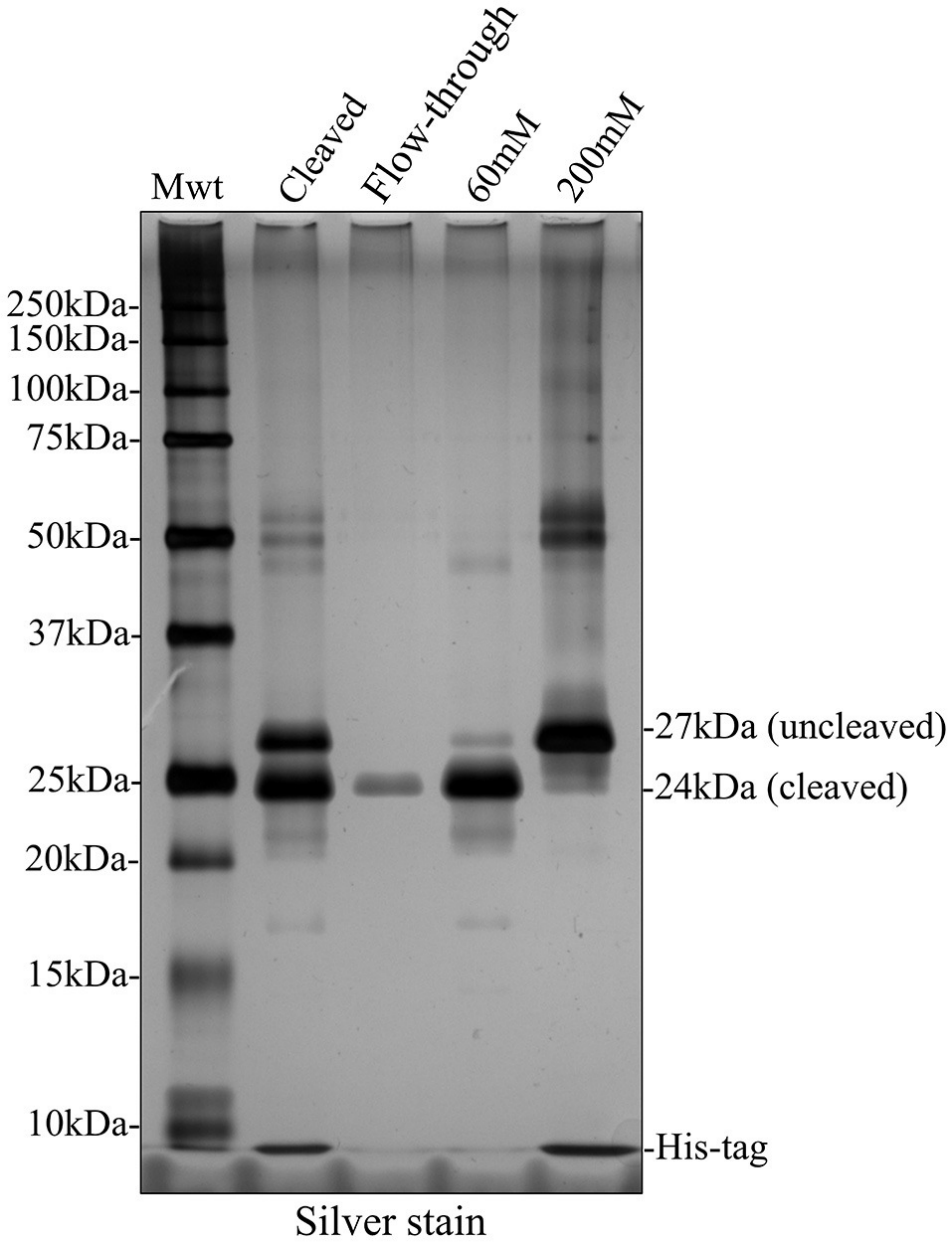
Silver stained SDS PAGE showing second round of nickel column purification to separate cleaved and uncleaved recombinant amelogenin. The bulk of cleaved recombinant amelogenin was eluted by 60 mM imidazole together with lower molecular weight contaminants and some uncleaved recombinant. The uncleaved amelogenin was eluted after increasing the imidazole concentration to 200 mM.

We attempted to remove the His-tag from recombinant amelogenin via proteolytic cleavage and then carried out a second round of affinity chromatography to remove any uncleaved amelogenin (still carrying the His-tag), free His-tag and the cleavage enzyme itself. Following analytical SDS PAGE, it was clear that that cleavage was only around 50% efficient at either pH 8 or 9 (Figure 5A and Supplementary Figure 1). In an attempt to purify cleaved from uncleaved His-tagged recombinant amelogenin, the sample was applied to the nickel column in 20 mM imidazole buffer after the cleavage step. Both cleaved and uncleaved recombinant amelogenins (without and with the His-tag, respectively) were retained on the column with very little cleaved recombinant appearing in the column flow through (Figure 5B). Increasing concentrations of imidazole buffer were then used in further attempts to optimize separation of cleaved from uncleaved His-tagged recombinant amelogenin via a 50–400 mM imidazole stepped gradient. However, low molecular weight contaminants and a trace of uncleaved recombinant amelogenin co-eluted with the cleaved recombinant amelogenin even at the lowest imidazole concentration employed (Figure 5B). Uncleaved recombinant and free His-tag were eluted using >200 mM imidazole. The cleavage enzyme, which is also His-tagged, has a molecular weight of 22 kDa and theoretically elutes at high imidazole concentration though it was not present in high enough concentrations to be detected here. Based on these data, we chose 60 mM imidazole as the optimum concentration to use for elution in subsequent experiments (Figure 6) as it would be expected to give a better yield than 50 mM imidazole without eluting appreciably more uncleaved contaminant.

### Alternative strategy avoiding a second round of nickel column purification: preparative SDS PAGE

**Figure 7:**
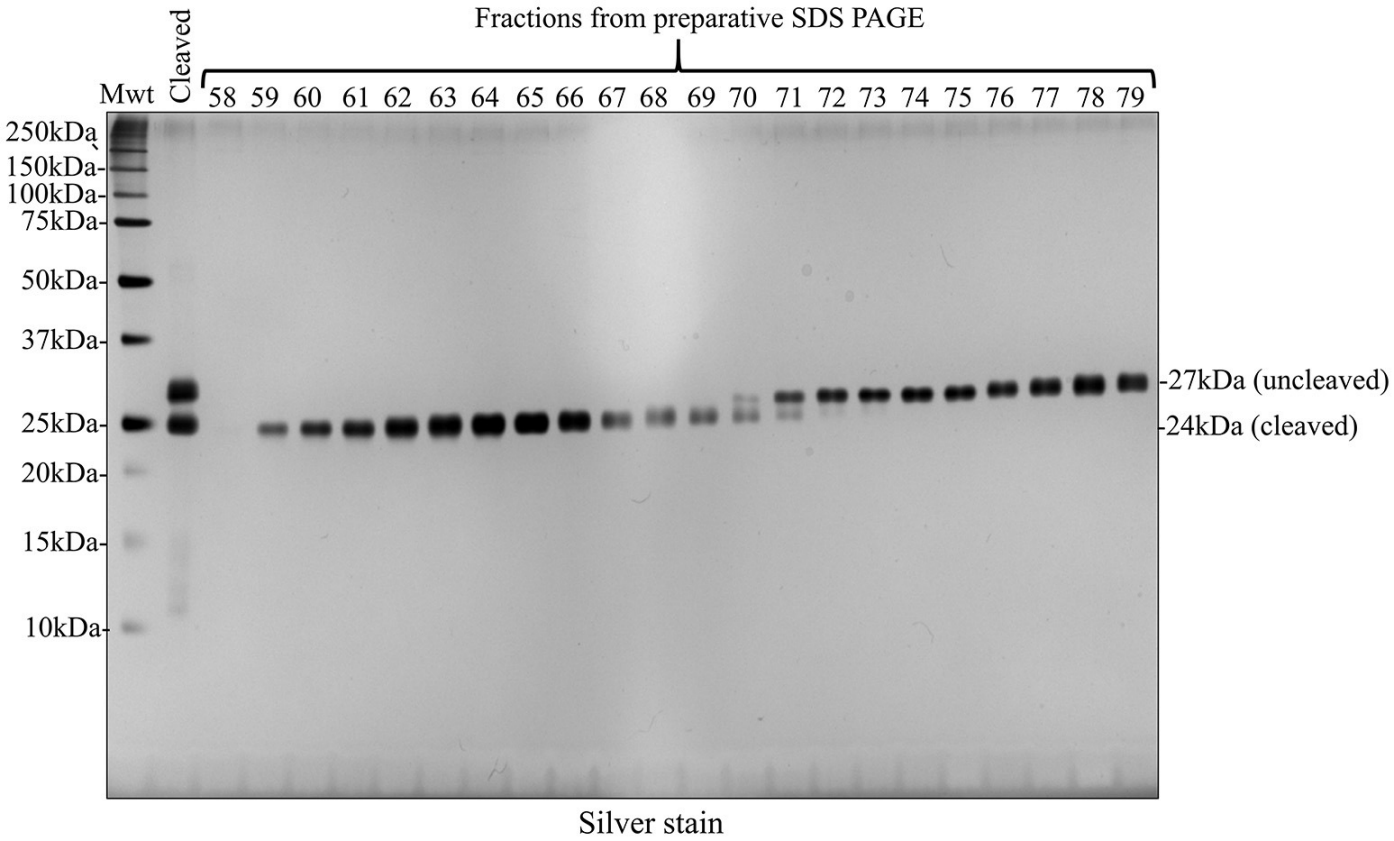
Preparative SDS PAGE purified cleaved recombinant amelogenin to single band purity on silver stained SDS PAGE analytical gels. Fractions 58–79 collected during preparative SDS PAGE of the HRV3C cleavage reaction mixture contained the 24 kDa cleaved and 27 kDa uncleaved recombinant amelogenins. Enhanced resolution of cleaved recombinant amelogenin was achieved compared with nickel column chromatography shown in Figures 5B, 6.

Given that a second round of nickel column purification failed to provide cleaved recombinant amelogenin at single band purity, we pursued an alternative strategy using preparative SDS PAGE. Fractions collected from preparative SDS PAGE were characterized using analytical SDS PAGE and silver staining (Figure 7). Using this method, cleaved recombinant amelogenin (without His-tag), migrating at 24 kDa, was completely separated from the uncleaved recombinant migrating at 27 kDa and any lower molecular weight contaminants. These were collected in earlier fractions (data not shown).

## Discussion

The production of recombinant proteins is essential to the study of protein function and particularly in the case of amelogenins, which are difficult to purify from native tissue due to their aggregative behavior, multiplicity of isoforms and their degradation products and scarcity of material in the most commonly used animal models. In addition, without recourse to recombinant expression, it would be almost impossible to study the effects of specific mutations on amelogenin behavior.

Despite much effort to obtain highly purified recombinant amelogenin using His-tag affinity chromatography we were unable to produce recombinant amelogenin of single band purity when analyzed by silver stained SDS PAGE. Apparent single band purity on SDS PAGE can be achieved by reducing sample load or by employing less sensitive gel staining techniques such as Coomassie Blue but this merely places any contaminants below the limit of stain detection. SDS PAGE is a standard technique for assessing degrees of protein heterogeneity in sample mixtures but additional analyses such as isoelectric focusing or mass spectroscopy would be needed to confirm absolute purity. With that caveat in mind, our goal was to produce recombinant amelogenin exhibiting single band purity on silver stained SDS PAGE gels. The challenges were twofold: (i) removing contaminating bacterial proteins and (ii) separating contaminating His-tagged recombinant amelogenin from His-tag-free recombinant amelogenin following His-tag cleavage, which is <100% efficient.

In an attempt to reduce contamination with bacterial proteins, we employed a previously described acetic acid extraction technique that reportedly produces recombinant amelogenin from *E. coli* at >95% purity in a single purification step (Svensson Bonde and Bulow, 2012). This technique is based on the simple premise that *E. coli* proteins are insoluble in 3% acetic acid at 80°C whereas amelogenin is soluble under these conditions. In our hands, the technique certainly provided a fraction highly enriched with recombinant amelogenin but this still contained contaminants easily detectable on silver stained SDS PAGE. Nevertheless, this initial acetic acid extraction provided a greatly enriched and “cleaner” sample for subsequent affinity chromatography on nickel columns. This is important as *E. coli* express a number of proteins (e.g., Fur, Crp, ArgE, SlyD, GlmS, GlgA, ODO1, ODO2, YadF, and YfbG) that are commonly co-purified when using immobilized metal affinity chromatography due to their metal chelating properties or clusters of exposed histidine residues (Bolanos-Garcia and Davies, 2006). Analytical SDS PAGE followed by silver staining showed that nickel column chromatography removed some of the residual contaminating protein from the initial acetic acid extract. Silver staining is highly sensitive but is non-quantitative so attempts were not made to quantify the efficiency of this purification step using gel densitometry.

Our data showed that His-tag cleavage of recombinant amelogenin by HRV3C protease was far from 100% efficient. This is in agreement with a previous report using rTEV protease to remove His-tags from recombinant amelogenin (Taylor et al., 2006). In some cases, cleavage efficiency was as low as 50% which significantly impacted on final yield. Poor cleavage efficiency may be due to steric hindrance blocking access of the enzyme to the cleavage site (Waugh, 2011). Amelogenin shows a great propensity to aggregate at physiological pH and temperatures (Moradian-Oldak et al., 1994, 1995, 1998; Simmer et al., 1994; Tan et al., 1998; Wiedemann-Bidlack et al., 2007; Aichmayer et al., 2010) and self assembles via N-terminal and/or C-terminal interactions to form higher order structures (Paine and Snead, 1997; Moradian-Oldak et al., 2000; Paine et al., 2000, 2003; Fang et al., 2011; Wald et al., 2017). We therefore hypothesized that amelogenin-amelogenin interactions may hinder HRV3C accessing the His-tag cleavage site. However, carrying out cleavage at 4°C (data not shown) and increasing the pH to 9.0, (both expected to favor amelogenin monomerization) had no effect (Supplementary Figure 1). Finally, we increased the cleavage time to 48 h but again this did not improve cleavage efficiency (data not shown).

Accepting the fact that His-tag cleavage was not 100% efficient, we subjected the cleaved sample (containing recombinant amelogenin with and without His-tags, free His-tag, cleavage enzyme, and any other residual contaminant) to a second round of nickel column affinity chromatography. In theory, the cleaved recombinant amelogenin should have little affinity for the column after removal of the His-tag ligand and would be easily collected in the flow through or at low imidazole concentration while the uncleaved recombinant amelogenin, free His-tag and HRV3C cleavage enzyme (which is itself His-tagged) would remain bound to the nickel column. However, cleaved recombinant amelogenin still exhibited significant affinity for the nickel column. This may be due to the high histidine content of amelogenin which includes a tri-histidine domain (Snead et al., 1985) which can cause non-specific binding confounding His-tag purification (Schmitt et al., 1993; Bornhorst and Falke, 2000). The cleaved recombinant amelogenin could be partially eluted with 50 mM imidazole but stepwise increases in the imidazole concentration resulted in the elution of more uncleaved recombinant amelogenin (Figure 5B). This suggested that several conformational isoforms of the cleaved recombinant amelogenin were present, each having a different affinity for the nickel column. The real problem however was that uncleaved recombinant amelogenin began to elute as the imidazole concentration approached 60 mM. This meant that eluting the cleaved recombinant amelogenin with 60 mM imidazole to maximize yield would result in the co-elution of contaminating uncleaved recombinant amelogenin. Even elution at 50 mM imidazole resulted in the co-elution of readily detectable uncleaved recombinant amelogenin.

**Figure 8:**
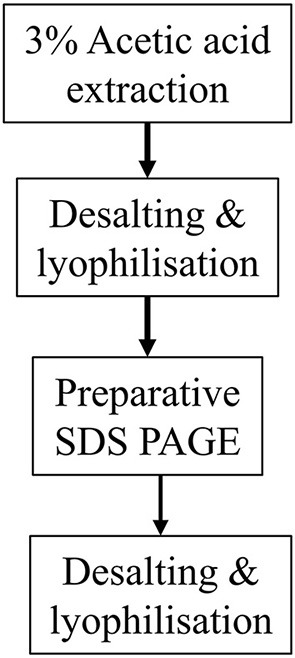
Proposed methodology using preparative SDS PAGE purification of His-tag free recombinant amelogenin.

To solve this problem we decided to employ preparative SDS PAGE rather than using nickel column affinity chromatography. This method was able to separate cleaved recombinant amelogenin from all contaminants to single band purity on analytical silver stained SDS PAGE. Substituting nickel column affinity purification with preparative SDS PAGE reduces the number of steps and leads to a much purer end product. Indeed, the excellent resolving power of preparative SDS PAGE, coupled with the acetic acid extraction technique makes the need for nickel column purification of His-tagged recombinant amelogenin redundant. We suggest that recombinant amelogenin without any His-tag could be purified to single band purity using preparative SDS PAGE (Figure 8). In comparison to other more costly and labor intensive methods, using the acetic acid extraction procedure in combination with preparative SDS PAGE provides an economical and speedy method for generating recombinant amelogenin. The lack of an N-terminal His-tag also eliminates any unwanted amino acids at the N-terminal remaining after His-tag cleavage. We are currently re-engineering our recombinant amelogenin vectors to remove the His-tag to take full advantage of the methodology described.

The preparative electrophoretic equipment employed here is not restricted to purifying proteins using SDS PAGE which separates proteins based on their molecular size. Separations can also be achieved using native PAGE buffer systems where proteins, in the absence of SDS, are separated based on their net charge which in turns depends on pH. The manufacturer's instructions provide a range of buffer formulations covering the pH range 3.8–10.2 which can be further adapted by the addition of chaotropic agents and uncharged detergents to aid protein solubility. Although, this additional flexibility is not required in the application reported here, it may prove useful if using preparative electrophoresis for other application such as purifying native enamel proteins extracted from developing enamel.

## Author contributions

SB, JK and CG made substantial contributions to the conception and analysis of data; drafted the paper and/or revised it critically for intellectual content; provided final approval of the version to be published; agree to be accountable for all aspects of the work in ensuring that questions related to the accuracy or integrity of any part of the work are appropriately investigated and resolved. CG acquired the data.

### Conflict of interest statement

The authors declare that the research was conducted in the absence of any commercial or financial relationships that could be construed as a potential conflict of interest.
