# Supplementary figures and images for: Preparative SDS PAGE as an Alternative to His-Tag Purification of Recombinant Amelogenin
Source: Front Physiol. 2017 Jun 16;8:424. doi: 10.3389/fphys.2017.00424 (PMC5472695; doi:10.3389/fphys.2017.00424)

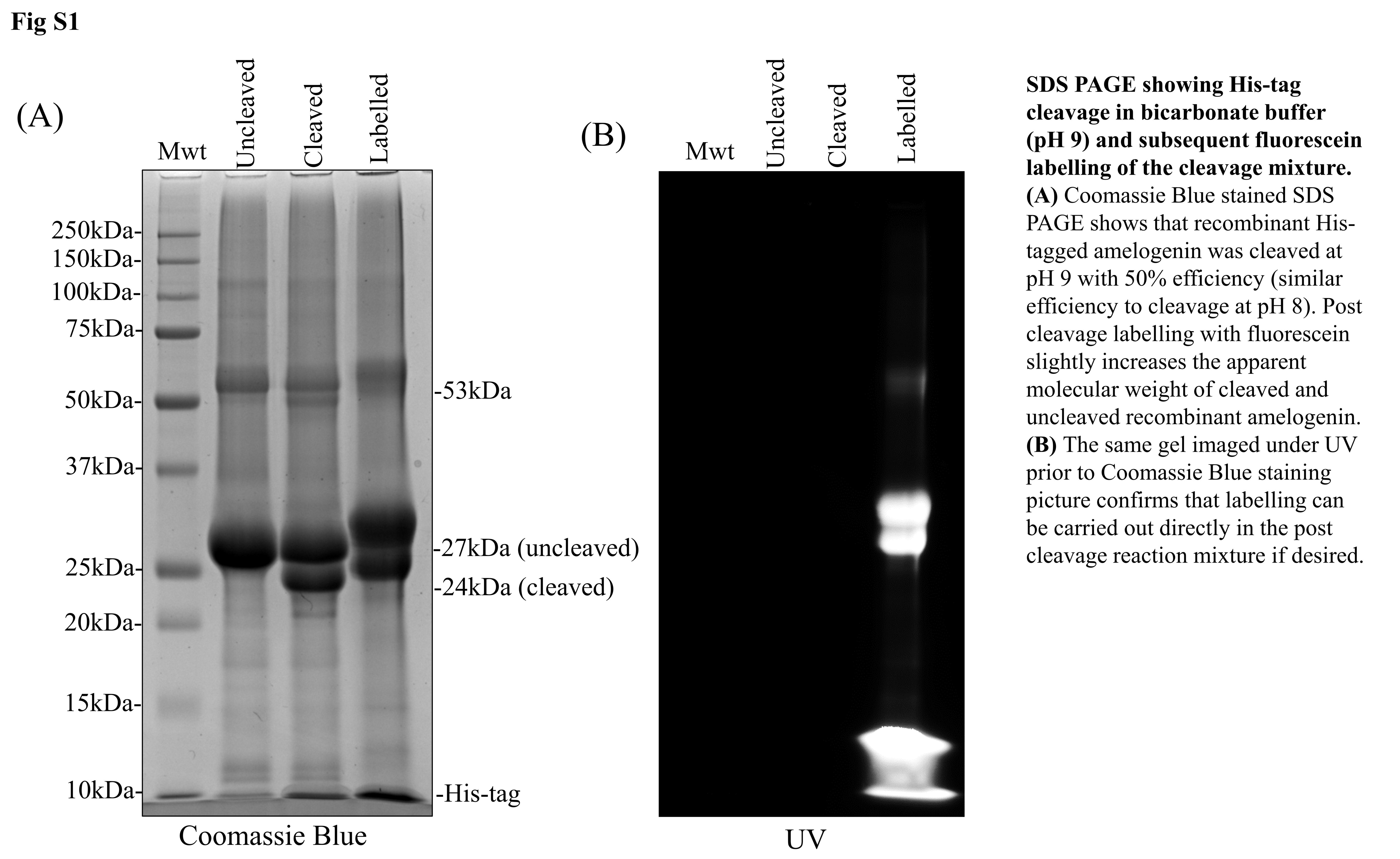

Supplement: Supplementary file 1 [file Image1.JPEG]
